# Medical and psychological consequences of rape among survivors during armed conflicts in northeast Ethiopia

**DOI:** 10.1371/journal.pone.0278859

**Published:** 2022-12-12

**Authors:** Lebeza Alemu Tenaw, Mulugeta Wodaje Aragie, Alemu Degu Ayele, Tsion Kokeb, Nigus Bililign Yimer

**Affiliations:** 1 School of Public Health, College of Health Sciences, Woldia University, Woldia, Ethiopia; 2 School of Midwifery, College of Health Sciences, Woldia University, Woldia, Ethiopia; 3 Department of Midwifery, College of Health Sciences, Debre Tabor University, Debre Tabor, Ethiopia; Chulabhorn Royal Academy, THAILAND

## Abstract

**Background:**

Rape is the most common act of violence against women during wartime which is considered interpersonal, social and political violence because survivors usually suffer from stigma and discrimination in the community. Sexual violence is a serious threat to women’s health. The psychological and medical consequences of rape during the conflict period are not well documented. Therefore, this study investigated the psychological and medical consequences of rape among survivor in the northern Ethiopia conflict, which occurred since 2020–2022.

**Methods:**

A retrospective cross-sectional survey supplemented with a qualitative data was conducted among survivors of rape recorded until June 2022. Health institutions that provide maternal and child health services in the study area were included. All rape victims who received medical care following the incident were included. Victims who were found in active war areas or rape care recorded before wartime were excluded. To understand the experience of raped women’s psychological consequences related to sexual assault we conducted 23 in-depth interviews. Thematic analysis was used to conduct qualitative interpretation.

**Results:**

The mean age of the participants was 31.66 (SD ± 20.95) years. One-third of 92(33.9%) of the survivors were diagnosed positive for sexually transmitted infections. Chlamydia 54(58.4%) and HIV 32(34.8%) were the most frequently diagnosed infections. Among the rape survivors, one-tenth 29(10.7%) of them were positive for pregnancy, and induced abortion was done for 13 (44.8%) women who got pregnant due to sexual assault. The armed groups not only have sexual interests but inhumane individuals and consider rape as their way of expressing abjection to civilians. Survivors of raped women are confronted with social rejection and exclusion in the community that aggravates the traumatic process. Because of shame and fear, rape survivors often do not seek help but have to be offered support proactively. The victims claimed that they didn’t able to return to their previous life and considered their future in peril.

**Conclusion:**

Conflict has a multidimensional devastating life effect, especially on women’s health. The victims experienced many physical and psychological consequences. Hence, resolving conflicts with peaceful discussion has numerous benefits for civilians.

## Introduction

The tragic and bloody northern Ethiopia conflict started on November 3, 2020, with a well-prepared nightly assault of the Tigray Peoples Liberation Front (TPLF) forces on the federal army based in Tigray Regional State, which expanded to Amhara region since June 2021, where major sexual abuses on local inhabitants were perpetrated [1, 2].

Wartime sexual violence is a devastating form of aggression committed against the civilian population during armed conflicts which remains one of the most inhumane characteristics of modern armed conflicts [3]. Although more men lose their lives during armed conflict, women and girls are disproportionately affected by sexual and gender-based violence including rape, which is a myriad debilitating consequence of war [3, 4].

Rape is defined as one person forcing another, without this person’s consent to have intercourse or other forms of sexual activity [5]. Conflict-related sexual violence refers to rape, sexual slavery, forced prostitution, forced marriage, and any other form of sexual violence perpetrated against individuals that is directly or indirectly linked to a conflict; which act as interpersonal and social violence because communities reject women from the social standard of living and erode individual relations and community and family structures [6, 7].

Thousands of women are being targeted by the deliberate tactic of using rape as a weapon in the northern Ethiopia conflict. Fighters affiliated with the Tigrayan People’s Liberation Front (TPLF) deliberately raped dozens of women, some as young as 14, in northeast Ethiopia [8].

Many of the survivors suffered severe and long-term physical and psychological damage, eventually, many victims remained hospitalized for up to three months after they were raped [9]. Its effect can be felt for a lifetime either through mothering unwanted children, in turn, can lead to high rates of unsafe abortion and maternal mortality, incontinence, or often diseases like syphilis, fistula, and HIV. Sexual health effects include unwanted pregnancies, complications from unsafe abortions, and sexually transmitted infections [3, 10].

It also does huge damage to families and communities and there is a real stigma and sense of shame around the women who have been raped, their husbands and families often find it very hard to accept the women back into the community after what’s happened to them. Mental health effects may include somatic complaints, depression, anxiety, alcohol and drug abuse, and committing suicide [11].

While these are wide-ranging and map onto the spectrum of civil, political, social, and economic rights violations, there remains a need to generate more in-depth investigations of the psychological and medical consequences subjected to the victims [3].

Women and girls are especially vulnerable to sexual violence during war and civil conflict, whether fighting while escaping from their homes, or even once inside camps for refugees or internally displaced people. World Health Organization (WHO) declares that eliminating all forms of violence against women and girls is critical for achieving the Sustainable Development Goals(SDGs) health targets [8, 12].

Victims of rape who become pregnant unintentionally may use risky methods to end the pregnancy, endangering their health and perhaps their lives. An important public health issue is unsafe abortion. Both moms and their newborn children are extremely vulnerable and have a higher risk of being excluded from the community. Even infanticide or other types of violence could be a risk for these kids [13].

To the best of our knowledge, the psychological and medical consequences of rapes during the invention of TPLF in the Amhara region are not well documented; and rehabilitation and resettlement programs fail to address the psychological and medical health needs of sexually abused survivors.

Additionally, this study has importance for survivors to access medical and psychological health services as their need which encourages humanitarian organizations to need to design protocols that take into account the medical, psychological, and social impact of all types of sexual violence. The findings of this study will provide input for emergency humanitarian interventions and help the government, and other stakeholders in designing empirical and evidence-based management guidelines for victim women; to design appropriate prevention strategies for medical complications related to rape. Last but not the list it will serve as baseline evidence for further investigation in the area by other researchers.

## Methods

### Study design and setting

A retrospective cross-sectional survey supplemented with the qualitative study will be conducted among victims of rape recorded until June 2022 since the invention of the Tigray People Liberation Front (TPLF) in Amhara region, Ethiopia.

This study was conducted in North Wollo and Wagihmera Zones, Northeast Ethiopia which was highly affected by the TPLF terrorist group. Amhara region is one of the eleven regional states found in Ethiopia which is invaded by the TPLF. North Wollo Zone is located about 521 km from Addis Ababa, the capital city of Ethiopia; and Waghemira Zone is an administrative zone in eastern Amhara having six districts with Sekota Town, the capital of the zone, is 720 km north of Addis Ababa, Ethiopia.

Quantitative data were collected from healthcare institutions that provide medical consultation, screening, and treatment of sexually transmitted infections (STIs), diagnosis and management of unwanted pregnancies, and other medical care for survivors. Health institutions that provide maternal and child health (MCH) services during the invasion and/or immediately after the area was freed from the invaders in the two zones of the Amhara region were visited. We followed a phenomenological approach to understand and describe raped women’s experiences during the armed conflict in northeast Ethiopia.

### Sample size and study population

According to an Amnesty International report, more than 70 women were raped in Nifas Mewcha town within 15 days stay of TPLF’s soldiers in mid-August 2021 [8]. Based on this data we estimated there may be around 349 raped women, who seek and access health service, in the two zones but only 271 victim women’s file was accessed in the health service during the study period.

We initially planned to conduct around 20 interviews to understand the experience of raped women’s medical and psychological consequences related to sexual assault. The recruitment was performed by the first author in collaboration with the local women and children’s affairs office. However, we eventually continued to do 23 in-depth interviews (IDI) to achieve maximum saturation.

All rape victims whether having received any medical care following or not during the TPLF invention were included. The study period was from May 15/2022 to June 5/2022. Victims who are found currently under invaded areas were not included. Cases of rape that occurred before this date were excluded. Rape is a criminal, aggressive and violent act to have sexual intercourse with a person without her consent.

### Sampling procedure

Health institutions that provide MCH services in the study area were included. The sample size in each health institution was proportionally allocated based on the number of survivors assessed in the selected health institutions. Two woredas from Wagihimera Zone (Sekota town, Gazegibla woreda,) and four Woredas from North Wollo Zone (Woldia Ketema, Meket Ketema, Raya Kobo woreda, Habru Woreda) were randomly selected. Survivors who did not visit health institutions were recruited to participate in a qualitative study using snowball sampling.

### Data collection instruments and procedures

Survivors’ age, marital status, level of education, and medical information were abstracted from medical records using a structured checklist modified from different literature focused on sexual abuse and its consequences during the armed conflict. The interviewer was recruited from the local area and selected based on previous experience of working with survivors and/or researching sensitive issues such as sexual violence. Following the quantitative portion, the principal investigator conducted in-depth interviews with one assistant from the respective health institution by using an in-depth interview guide. It explores the psychological consequences of the northern Ethiopia conflict on women, and a tape recorder was used to facilitate the conversation. During the data collection process, we follow the WHO and local COVID-19 prevention protocols.

Pre-testing and training for data collectors and supervisors were done to ensure the quality of the data. Data confidentiality, participant rights, informed permission, the purpose of the study, interviewing techniques, and questionnaire completion was the main topics of the training.

### Data quality control

Interviewers and data collectors were trained on research aims, methodology, recruitment, interviewing techniques, and ethics. In addition, daily debriefings were held with the interviewers during data collection. To assure the validity of the checklist tools and in-depth interview guide questionnaires we conducted a pretest.

### Variables and data analysis

Variables including age, marital status, education level, and time to medical care access (in terms of the delay from the incident of rape to seeking care) were assessed and presented through descriptive statistics. Medical consequences due to rape include sexually transmitted infections, traumatic pelvic pain, fistulas; postrape pregnancies and others were also reported in a table with their frequencies. Data were recorded and analyzed by using SPSS version 23 software.

All in-depth interviews were audio-recorded. Then, to get a feeling of being whole, we listened to the recorded audio and chose units or segments that were relevant to our research goals. The data were transcribed verbatim by two investigators separately and translated into English respectively. After comparing each translation and checking the consistency, the data were coded. The segments were the meaning units that made up the various components of the phenomenon’s essential structure. We listened to the data and reread our notes before categorizing everything into broad groups.

We regularly compared categories and looked for patterns and themes as they appeared during the investigation. We used our findings with the context to synthesize, modify, and structure our understanding of several thematic sections of the phenomenon. Thematic analysis was used to conduct qualitative interpretation.

#### Ethical considerations

The ethical clearance was obtained from the Institutional Review Board (IRB) of Woldia University with protocol number WDU/IRB001. A formal letter prepared by the university’s research and development office was given to the selected health institutions. Moreover written consent was obtained from each respondent and aware of their right to withdraw from the study at any time during the interview period. Written informed consent was obtained from a parent or guardian for those under 18 years old participants. Interviews were conducted in private rooms with only the interviewer, and confidentiality was assured. We identified all recorded interview files with a unique code, understandable only to the corresponding author. The dissemination of the finding was not to be referring to a specific respondent but the general source population.

## Results

### Socio-demographic characteristics of participants

A total of 271 rape survivors participated in the quantitative data. To supplement the study, 23 rape victim women participated in the in-depth interview for the qualitative study. The mean age of the participants was 31.66 (SD ± 20.95) years with a predominant age group of 19–30 years. Two-thirds of 180(66.4%) of the participants were rural residents. Additionally, more than a third of the 100(36.9%) of the participants were divorced (Table 1).

**Table 1:** Socio-demographic characteristics of rape among survivors during armed conflicts in Northeast Ethiopia, 2022 (n = 271).

Variable		Frequency	Percentage
Age of survivors	<19	37	13.7%
	19–30	104	38.4%
	31–40	86	31.7%
	>40	44	16.2%
Residence	Rural	180	66.4%
	Urban	91	33.6%
Marital Status	Not in union	99	36.5%
	Married	42	15.5%
	Divorced	100	36.9%
	Widowed	30	11.1%
Educational Status	No formal education	135	49.8%
	Primary education	59	21.8%
	Secondary education	71	26.2%
	Tertiary education	6	2.2%

### Characteristics of sexual assault

The majority of perpetrators raped the women at the victims’ homes when they stayed alone. Nearly three-quarters of 200(73.8%) of the survivors were sexually assaulted at their homes (Table 2).

**Table 2:** Features of sexual assault during armed conflicts in Northeast Ethiopia, 2022 (n = 271).

Variable		Frequency	Percentage
Number of the perpetrators	One soldier	174	64.2%
	Two soldiers	60	22.1%
	Three and above soldiers	37	13.7%
Place of assault	At victims home	200	73.8%
	Out of the victim’s home	71	26.2%
Presence of another person during the assault	Yes	48	17.7%
	No	223	82.3%

### Inhumane acts of the perpetrators which considered revenge

Violence against women and children has gained international recognition as a grave social and human rights violation which is more devastating when the perpetrators are armed forces. Rape is used as a systematic weapon of warfare with the aim to torture and humiliate the victims and their families, destroy the fabric of the community, which occurs in many conflicts and crises.

The survivors were blamed; some perpetrators invited HIV-positive armed men to have sex with healthy civilian women. The armed groups not only had sexual interests, but inhumane individuals and considered rape as their way of expressing abjection of the Amhara people.

*“I asked them to protect me from becoming pregnant and contracting a sexually transmitted infection*, *but they said that you are fortunately to have sexual contact with us and three troops interchangeably raped me for nine days*. *I feel they aimed to transmit communicable diseases through the community”*, said the victim.

The participants expressed that the perpetrators not only raped them but also they were scoffing at their damage, which shows their level of brutality against civilians. Survivors feel the perpetrators did not show any humbleness even for kids and the elderly.

*“Perpetrators pulled me like a sheep on my door even though I was bleeding profusely*. *They shot and killed my sister’s kid right in front of my eyes*. *When I saw this tragic situation*, *I couldn’t control myself”*, said another victim.

**Fig 1:**
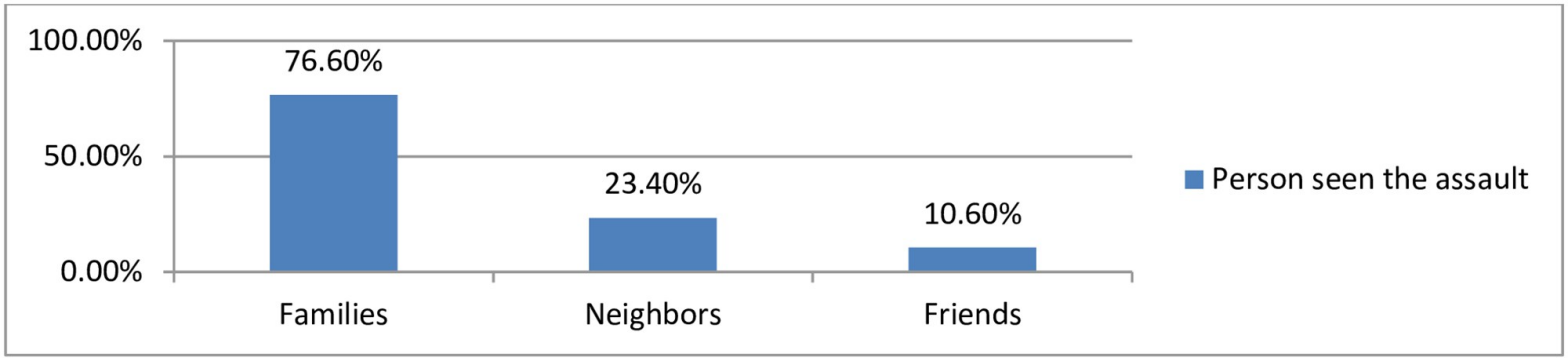
Individuals who have seen when the victims were assaulted during armed conflicts in Northeast Ethiopia, 2022 (n = 271).

The majority of the survivors 208(76.6%) blamed that they were sexually assaulted in front of their families (Fig 1).

### Community discrimination and social taboos attached to victims

Survivors of rape are almost always confronted with social rejection and exclusion that aggravate the traumatic process. Hence, it is of utmost importance to avoid conditions that increase stigma and exposure to public judgments of survivors of sexual violence. Survival of rape blamed the bad social response for their assault and was deeply traumatized by the negative response of the community. Even though the community is expected to support the victims when they need help, some members of the community scoff at the survivors and their children.

*“I am a pity on my neighbors because I expected them to save me from the junta*. *But no one came to help me*. *Throughout this upheaval*, *everyone in my neighborhood was aware of what was going on*, *yet no one came to help me*. *I’m sad because our community has borne yet another load for us”* said widowed victim.

Because of shame and fear, rape survivors often do not seek help but have to be offered support proactively. The true support of the rape victim comes when the communities consider the damage of the survivors as the community’s assault because the victim carries the whole burden of the community.

*“When certain members of the community saw me*, *they veiled me*. *They claimed she was the junta’s wife*. *I’m not able to interact with my neighbors as much as I used to do because I’m afraid they’ll gossip about me*. *Even I go to church on my own”* said another victim.

Some members of the community also perceived the raped women as the beneficiary of donors/government and saw the victims’ sexual assault as a benefit. They blamed the victims as they were raped without their consent.

*“I cannot get out of my bed and have been sleeping for 15 days and nights*. *I feel depressed*, *and contemplating suicide*, *but I ‘did not want to hurt anyone because they banter with us*. *Even when I came for this interview*, *the community claimed that I came to get benefits”* said the married victim.

### Medical consequences related to sexual assault

**Fig 2:**
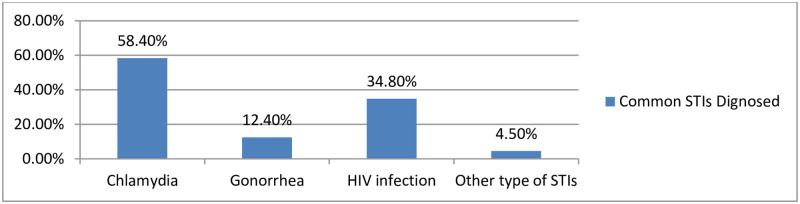
Sexually transmitted infections survivors exposed in Northeast Ethiopia, 2022 (n = 92).

**Fig 3:**
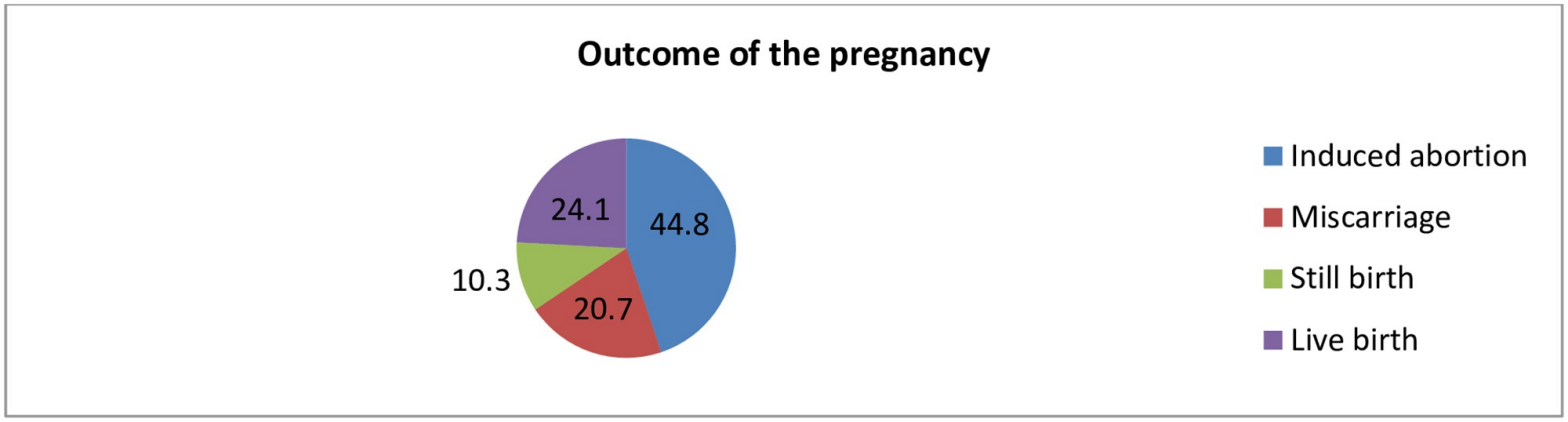
The outcomes of the pregnancy which occurred among rape victim women in Northeast Ethiopia, 2022 (n = 29).

Among the victims who had STIs, 54(58.4%) had chlamydia and 32(34.8%) of them were diagnosed with HIV infection (Fig 2). Alitle more than one-third of 92(33.9%) of the victims were diagnosed positive for sexually transmitted infections. Among the survivors, 29(10.7%) women were HCG positive for pregnancy (Table 3). Among pregnant women, 13 (44.8%) of them terminated the pregnancy through induced medical abortion (Fig 3).

**Table 3:** Medical consequences of rape among victim women during armed conflicts in Northeast Ethiopia, 2022 (n = 271).

Variable		Frequency	Percentage
Time of accessing health care	Within 72hrs	19	7.0%
	After 72hrs	252	93.0%
Pregnancy happened	Yes	29	10.7%
	No	242	89.3%
Vagina bleeding	Yes	40	14.8%
	No	231	85.2%
Vaginal discharge	Yes	60	22.1%
	No	211	77.9%
Result of any STI diagnosis	Positive	92	33.9%
	Negative	158	58.3%
	Not diagnosed with STI	21	7.75%
Urinary infection	Yes	41	15.1%
	No	230	84.9%
Genital injury	Yes	57	21.0%
	No	214	79.0%
Musculoskeletal pain	Yes	82	30.3%
	No	189	69.7%
Loss of consciousness	Yes	14	5.2%
	No	257	94.8%

### Psychological consequences related to sexual assault

Rape can have serious health consequences for women, exposing survivors to large-scale effect throughout their lives, ranging from sexually transmitted diseases such as HIV/AIDS to incontinence, from fistula to infertility and unwanted pregnancy to unsafe abortions.

*“They raped me not only on the genital but also rectal*. *I lost consciousness*. *My breast was beaten and infected as a result*. *I was also exposed to several infections of the urinary and reproductive organs*. *I can’t sit to pee because my genital and pelvic areas were injured*. *As a result of the assault*, *I was diagnosed with HIV positive”* said the divorced victim.

Victims’ work performance in the field may be severely reduced due to physical and mental health consequences. The victims claimed that they were not able to return to their normal lives. According to the survivors, their future is in peril. They claimed as they can’t seem to keep their attention any longer. The assault has an impact on the victims’ entire lives. The participant was scared that the sexual assault might happen again. They blamed that they were terrified when they saw someone wearing ranger clothes.

*“Since that day*, *I haven’t taken a single nap*. *My child woke up in the middle of his slumber since he didn’t forget what he saw*. *Throughout the night*, *I repeatedly check my doors*. *After that*, *I lost my attention; and can’t recall what I sell or purchase when I go to the market*. *My body also became a shrine on its own*. *I’m not able to return to my previous life”* said another divorced victim.

Survivors experience mental and psychological problems, like feelings of low self-esteem, anxiety, depression, eating disorders, sleep disorders, and suicide attempts. When they recall the incident, they fell sadly. Many victims claimed that they could not forget what was happened. They are terrified when visiting the place where they were raped. Nonetheless, one question arises in their minds: Who protects ladies from this heinous sexual abuse?

*“I was still concerned that they could return*. *I’m still unable to sleep*. *They anger me by acting like terror troops*. *I take a step forward and appeal to my God for relief*. *I recorded my age as 90 years when I went to get an ID card from my kebele because I’m expecting to leave as older when they come back*”, said another participant.

### Challenges to accessing medical care

Due to the problems to access health care, the majority 252(93%) of the survivors did not get health care within the first 72hrs (Table 3). Medical attention and specialized assistance for rape victims are instant responses to ensure the survivors’ health. The study participants claimed that there were no health institutions that deliver even emergency health services. Even after the invaders moved out from our village, the health institutions are not functional yet.

*“I had an HIV test and got two different results*. *One said positively*, *while the other replied negatively*. *I can’t check the final result of my health status because the health institutions are stocked out*. *Since then*, *my spouse has been sick*, *but we haven’t been able to get to the doctor for a checkup”*, said another victim.

**Fig 4:**
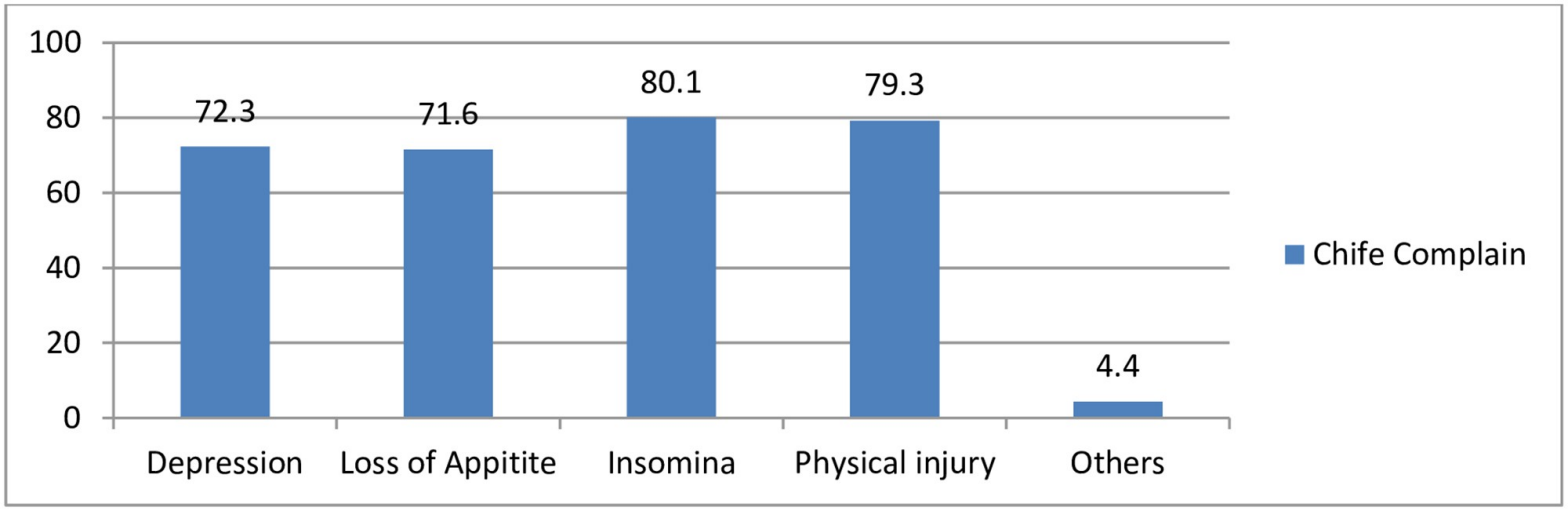
Common chief complains victims claimed in the health care provision in Northeast Ethiopia, 2022 (n = 271).

The main chief complaints the victims mentioned during their healthcare provision were insomnia 79.7% and physical injury 79.3% (Fig 4).

## Discussion

This study’s findings indicate that the consequences of sexual assault on women should be considered a major public health concern. Rape is linked to a variety of short- and long-term conditions, such as physical harm and disease, psychological symptoms, fial consequences, and death. According to the findings of this study, any woman of any age and educational level is exposed to sexual assault.

Various medical issues have been identified in rape victim women. Rape can result in the victim contracting a sexually transmitted infection (STIs). According to the current study, up to 43% of rape survivors were diagnosed with sexually transmitted infections during the northern Ethiopia conflict, which is lower than women who presented to the Harborview Medical Center in Seattle because of a recent sexual assault, have been found in up to one third (33.9%) of rape victims diagnosed with STTs infection [14]. The reason for this difference may be due to the characteristics of the perpetrators since in the current study participants were considered enemies by the armed forces.

Similarly, the current study’s findings found lower STI exposure than a study on raped women in the Democratic Republic of Congo, which revealed that 44% of rape victims were STI-positive [15]. This may be because the current study was assessed in an area where functional health institutions were not available and STI diagnostic kits were stocked out.

This study revealed that one-tenth (10.7%) of rape survivors were HCG positive in pregnancy, which is greater than a previous study’s findings where only about 5% of rapes result in pregnancy [16]. Similarly, the rate of pregnancy in this study was higher than the rate of pregnancy in the DRC study, when only 8.3% of the victims were pregnant [15]. The main reason for these finding differences may be the inaccessibility of contraceptives including emergency/post pills in the area where the current study covered.

The current study findings showed that 44.8% of pregnancies ended in induced abortion, which is lower than the 54.7% of pregnancies that ended in abortion in the DRC study. The findings do not also in line with those of studies conducted in the United States, which found an abortion rate of 1.9% to 5%. The difference between these results can be explained by the fact that the studies were conducted in two different contexts. In the current study, the participants considered the perpetrators as the enemy and didn’t intend to have a child with them [17–19].

The main consequence of rape on victims during their healthcare provision is insomnia 79.7% and physical injury 79.3%, which is in line with the finding studied in Los Angeles which stated as raped women experienced more symptoms of physical, and medically explained and psychological ill health than no victimized women [20]. Back pain (30.3%) and genital injuries (21.0%) were among the symptoms and illnesses reported by rape victims, which corresponded to the findings of the raped women-targeted study done in the other area [21].

According to the findings of the current study, 72.3% of the study participants claimed depression during their health care, which is consistent with the study finding that women who have been sexually assaulted are disproportionately depressed; sexually abused children had a 51% higher chance of developing depression as young adults [22]. This study’s findings also imply that children who are exposed to parental violence are at risk for long-term emotional and behavioral problems. Even if children are not abused personally, growing up in a home where their parents are violent puts them at risk, similar to reports released in other studies [17].

Qualitatively, the current findings showed that even though the community expected to support the victims when they shouted for help, some members of the community scoffed at the survivors and their children. Another study has supported this finding, which indicates that rape victims have difficulty interacting with others, which might negatively affect their social ties. Sexual assault creates a bad psychological impression on the victim’s friends, family, and partners [23].

### Limitations of the study

The retrospective chart review study was challenged with missing data in the medical record and some of the information written in the medical record didn’t follow the appropriate protocols. The other limitation of this study was selection bias since the study used all victims registered in the medical record.

## Conclusions and recommendations

Most of the medical consequences related to rape were STIs including HIV/AIDS, unwanted pregnancies with their corollaries like abortion, and genital injuries. Additionally, the psychological consequences of rape were expressed as poor performance at work, loss of sleep, loss of appetite, and weak social interaction.

The health sector stakeholders better establish well-functional health institutions with the necessary diagnostic kit, provide appropriate medical and psychological interventions, design follow-up systems to access the victims, search for hidden victims to provide health care; and minimize the consequences related to the assault. The local community is also better to share victims’ burden as self since their assault is not only related to them and discourages social taboos and discrimination. Hence, resolving conflicts with peaceful discussion has numerous benefits for civilians.

## Abbreviations

HIV: Humane Immune Virus
IDI: In-Depth Interviews
IRB: Institutional Review Board
MCH: maternal and child health
RDS: Respondent-Driven Sampling
SD: Standard Deviation
SDGs: Sustainable Development Goals
SPSS: Statically Package for Social Science
STIs: Sexually Transmitted Infections
TPLF: Tigray Peoples Liberation Front
